# Mitochondrial DNA sequence context in the penetrance of mitochondrial t-RNA mutations: A study across multiple lineages with diagnostic implications

**DOI:** 10.1371/journal.pone.0187862

**Published:** 2017-11-21

**Authors:** Rachel A. Queen, Jannetta S. Steyn, Phillip Lord, Joanna L. Elson

**Affiliations:** 1 Institute of Genetic Medicine, Newcastle University, Newcastle-upon-Tyne, United Kingdom; 2 School of Computing Science, Newcastle University, Newcastle-upon-Tyne, United Kingdom; 3 Centre for Human Metabonomics, North-West University, Potchefstroom, South Africa; Seoul National University College of Medicine, REPUBLIC OF KOREA

## Abstract

Mitochondrial DNA (mtDNA) mutations are well recognized as an important cause of inherited disease. Diseases caused by mtDNA mutations exhibit a high degree of clinical heterogeneity with a complex genotype-phenotype relationship, with many such mutations exhibiting incomplete penetrance. There is evidence that the spectrum of mutations causing mitochondrial disease might differ between different mitochondrial lineages (haplogroups) seen in different global populations. This would point to the importance of sequence context in the expression of mutations. To explore this possibility, we looked for mutations which are known to cause disease in humans, in animals of other species unaffected by mtDNA disease. The mt-tRNA genes are the location of many pathogenic mutations, with the m.3243A>G mutation on the mt-tRNA-Leu(UUR) being the most frequently seen mutation in humans. This study looked for the presence of m.3243A>G in 2784 sequences from 33 species, as well as any of the other mutations reported in association with disease located on mt-tRNA-Leu(UUR). We report a number of disease associated variations found on mt-tRNA-Leu(UUR) in other chordates, as the major population variant, with m.3243A>G being seen in 6 species. In these, we also found a number of mutations which appear compensatory and which could prevent the pathogenicity associated with this change in humans. This work has important implications for the discovery and diagnosis of mtDNA mutations in non-European populations. In addition, it might provide a partial explanation for the conflicting results in the literature that examines the role of mtDNA variants in complex traits.

## Introduction

Mitochondria are double membrane-bound organelles found in eukaryote cells. They have a number of roles within the cells, including providing most of the cells energy through oxidative phosphorylation (OXPHOS). Additionally, they are involved in apoptotic cell death, the control of calcium concentration and other cellular pathways [1]. Mitochondria operate under the dual control of the nuclear genome and mtDNA. The human mitochondrial genome or mitochondrial DNA (mtDNA) is a 16,569 bp circular strand of DNA. The mitochondrial chromosome contains 37 genes, which encode for 13 proteins, 22 tRNA molecules and 2 ribosomal RNA molecules. The 13 proteins form the core of four of the five enzymes that carry out OXPHOS. The mtDNA has several unusual features: mitochondria and mitochondrial DNA are inherited strictly through the maternal line [2], so the mtDNA does not undergo bi-parental recombination. MtDNA is found in high copy number ranging from several hundreds to thousands per cell. Within a single cell, mtDNA copies may all be same (*homoplasmic*) or different (*heteroplasmic*) [1]. In humans, pathogenic mutations are normally seen as heteroplasmic variants; that is there is a mixture of wild-type and mutant mtDNA [1, 3]. MtDNA mutations do not exhibit a clinical phenotype until they are present of the majority of the mtDNA molecules within the cells.

Mutations of mtDNA are an important cause of inherited disease; over 250 pathogenic mutations, deletions and re-arrangements have been identified in humans [1, 3]. Mitochondrial diseases predominantly affect tissues with a high energy demand; so, neurological and muscular symptoms are common. Mitochondrial diseases are the most common cause of inherited metabolic diseases within new-borns [4]. In the UK adult population, the minimum prevalence rate for mtDNA mutations is estimated to be 1 in 5,000 [5]. Despite excellent estimates of disease frequency in some, particularly European, populations [6, 7], the spectrum of mutations that cause disease is less well understood in others, such as Black Africans. This leads to less accurate assessment of the impact of mtDNA disease in these populations [8, 9].

With maternal inheritance, mtDNA evolution has resulted in the emergence of distinct lineages called haplogroups. While the high level of mtDNA variation seen within human populations is useful to study population histories [10], it has made the identification of disease causing mutations difficult [7, 11]. Mitochondrial variation in humans has been extensively studied and has been compiled in MITOMAP database (http://www.mitomap.org) [12], Phylotree (http://www.phylotree.org/) [13] and tRNA-MAMIT online resource [14]. tRNA-MAMIT provides an online resource comparing single representative mt-tRNA sequences from 150 mammals which have been aligned to the revised Cambridge Reference Sequence (rCRS) genome [15]. The specific mitochondrial background (or haplogroup) may influence the expression of a disease causing mutations [16–18]. In human populations, there is some evidence suggesting that additional polymorphisms can either repress or compensate for pathogenic mutations [19, 20]; this has been proposed as an explanation for how different human populations differ in both the spectrum of mtDNA mutations and the variable symptoms present in patients [16].

Many of the mtDNA point mutations reported to cause disease in humans are located in one of the 22 mt-tRNA’s, which are encoded by mtDNA. Although mt-tRNA genes make up just 10% of the mitochondrial chromosome, ~60% of adult patients with mtDNA disease have a mutation within these genes [3]. Each mt-tRNA gene is approximately 70–75 base pairs in length. The secondary structure of tRNA molecule is described as clover leaf which is made of four domains, namely: the acceptor (acc) stem; dihydrouracil (D) stem/loop; the anticodon (ac) stem/loop; and the T Ψ C (T) stem/loop. The four stems in the tRNA molecule primarily exist as Watson-Crick base pairs, which maintain the clover leaf structure [21].The clover leaf structure folds into a 3D L-shaped molecule which, in turn, is held together by long range tertiary interactions. These tertiary interactions between bases stabilise non Watson-Crick base pairings in the stem regions of the tRNA molecule. mt-tRNA is variable and significantly different to genomic tRNA both in terms of the size of the D and T domains and nucleotide sequence. Although there are some differences between the mitochondrial encoded and nuclear encoded tRNA’s, a detailed study of mammalian mt-tRNA has shown that many generalisations about the mt-tRNA molecules still apply [21].

The most common point mutation seen in Caucasian European patients with mitochondrial disease is called m.3243A>G. [22, 23]. Multiple phenotypes are associated with m.3243A>G, including Maternally Inherited Diabetes and Deafness (MIDD) and Mitochondrial Encephalomyopathy, Lactic acidosis, and Stroke-like episodes (MELAS). 80% of MELAS patients carry the m.3243A>G mutation [24]. The nucleotide at position 3243 within the mtDNA molecule, equates to nucleotide 14 within the mt-tRNA-Leu(UUR) molecule. This nucleotide is predicted to have a tertiary reaction with nucleotides at positions 8, 14 and 21 [21]. The mutation therefore disrupts the tertiary folding structure of the mt-tRNA-Leu which: reduces the capacity for amino-acylation [25], prevents the termination of translation by the 16S ribosomal subunit [26]; and, inhibits the methylation of the U in wobble position of the mt-tRNA molecule [1, 27]. Critically, this variant is rarely reported in Black African patients with mtDNA disease [28]

One way to explore the importance of haplogroup context on the expression of mtDNA disease is to look for mutations in other animals which are disease-causing in human in the absence of disease in the animal. Then to look for possible compensating mutations where disease is not evident in the non-human animal grouping. For this strategy to be valid it is important to demonstrate mitochondrial disease in other species. A deletion in the mt-tRNA-Tyr gene in position 5304, associated with sensory ataxic neuropathy, similar to human myopathies, has been observed in golden retrievers [29] this provides a clinical example that these variants can have an effect in other mammals. It would be expected that a non-domestic animal would have severely reduced fitness [30]. Additionally it is important to be able to examine a substantial number of sequences, some prior work in this area has used limited numbers of species only considering a single individual per species [31]. Perhaps most importantly clinically validated criteria need to be applied to variants reported as mutations to ensure that they are truly causative of primary mitochondrial disease [3, 7, 32], such clarity on the criteria for pathogenicity was not in place at the time of prior publications on this topic [30, 31].

In this investigation, we studied human disease-associated variants of mitochondrial mt-tRNA-Leu (UUR) gene in other chordates to further the understanding of sequence context in modulating disease expression. This study looked in detail at mt-tRNA-Leu (UUR) in all chordates, for which there are at least 30 complete mtDNA sequences listed in GenBank. We focused on the m.3243A>G mutation because this is both the most common point mutation know cause of mitochondrial disease and the best studied. We have found that this mutation does occur in quite a few animals and have identified several likely compensatory mutations. We also present a comprehensive survey of all the mt-tRNA’s variants associated with disease in humans found to be fixed in other species, then consider those that are clinically proven as pathogenic [32]. We used publically available sequence resources that have grown massively over the last decade. This work supports the assertion that the mutational potential of mt-tRNA’s is strongly contained by sequence context [30]. As such, this study helps us to better appreciate the importance of haplogroup contexts in the penetrance and expression on disease causing variants. In the last few years the importance of this type of understanding the variable penetrance of mtDNA mutations, and its impact the discovery and diagnosis of disease causing mutations, especially in populations that have been less well studied [8, 9] has become increasing clear.

## Material and methods

### Reference sequence

The revised Cambridge Reference Sequence (rCRS) [15], was used as a reference sequence used in this investigation (GenBank record NC_012920.1). The position and order of mt-tRNA genes within the mitochondrial chromosome varies between species therefore in this study, all genome positions refer to the equivalent nucleotide position within the rCRS rather than the actual nucleotide position for the individual species. Nucleotide positions within the mt-tRNA molecule itself are also included.

### Identification of SNPs associated with disease

A list of known or suspected pathogenic SNPs, a list of SNPs within mt-tRNA-Leu (UUR) gene, associated with disease, was compiled from the MITOMAP and tRNA-MAMIT databases.

Additionally, a literature search was performed to look for SNPs which had not been added to either of the databases. The PubMed search query “Mitochondrial tRNA” was used to look for any articles published between 2014 and 2015 which reported disease association with SNPs. The likelihood of a role in pathogenicity of each disease associated SNP was assessed the scoring algorithm described by Yarham et al was applied [32].

### Extraction of mt-tRNA sequences from GenBank files

Complete mitochondrial genome sequences were downloaded from NCBI in GenBank format [33]. Each record within the file contains the species name and positions of the individual mt-tRNA genes within the mitochondrial sequences, in the annotation section of the record. Custom python scripts were used to extract mt-tRNA sequences from GenBank files containing complete mtDNA sequences. The Biopython SeqIO module was used to extract the gene locations for each mt-tRNA genes within each individual record. A search for the term "product" within the record qualifiers object was used executed to capture the start and end locations of all mitochondrial genes. The mt-tRNA sequences were extracted from the file using the gene locations contained in the sequence annotations. Each mt-tRNA sequence, for each species studied, was saved in a separate FASTA file. The product names given to the sequences correspond to those used within the rCRS sequence. As there are two mitochondrial mt-tRNA-Leu and tRNA-Ser genes, the sequences were then compared to the rCRS using the Biopython pairwise module. The pairwise scores were used to determine which of the two genes were present and separate the genes into the correct file. When it was not possible to distinguish between the sequences based on pairwise score, the sequences were placed into a separate temporary file for visual inspection.

### Gene alignment

The mt-tRNA sequences from the different species were aligned to the complementary mt-tRNA sequence from the rCRS and these alignments were then used to look for nucleotide changes at specific locations associated with disease in humans.

The mt-tRNA gene sequences from the rCRS genome were added to the previously generated files. ClustalW was used to create the alignments of the sequences [34]. The Biopython AlignIO module was used to create the alignments for each file.

A Python script was written using the Biopython module AlignIO which was used to detect polymorphisms at specific positions within the alignments. The script allowed for gaps within the alignment by converting the nucleotide equivalent in the rCRS mt-tRNA molecule to the nucleotide position within the alignment.

Sequences were used to study 33 disease associated variants within tRNA-Leu (UUR) gene.

The mitoseq section of MITOMAP provides executable search terms to retrieve all available mitochondrial genome sequences in GenBank (http://www.mitomap.org/bin/view.pl/MITOMAP/MitoSeqs). To collect sequence data for study the search "Complete non-human mtDNA genomes" was executed, to create a file containing all non-human mitochondrial genomes. The contents of this file was analysed using a script, which used the Biopython SeqIO module to generate a list of species to study. Species were selected if they met the following criteria:

1. 30 or more complete mitochondrial sequences were available within GenBank for the species. This threshold was set to allow the study of variation within species as well as between the species and humans to be conducted.
2. The species belonged to the phylum *Chordata*. The majority of mitochondrial diseases are neuromuscular therefore only species, possessing a central nervous system were chosen for study. Species which met these criteria are shown in Table 1.

**Table 1:** The 33 chordate species with greater than 30 complete mtDNA sequences used in the initial phase of the study.

Species	Common Name	Number of Sequences before QC	Number of Sequences after QC
*Anguilla anguilla*	European Eel	55	54
*Anguilla rostrata*	American Eel	51	51
*Balaenoptera physalus*	Fin Whale	154	154
*Bison bison*	Bison	34	34
*Bos grunniens*	Yak	83	83
*Bos taurus*	Cow	275	275
*Canis lupus familiaris*	Dog	391	391
*Clupea harengus*	Alantic Herring	100	100
*Coregonus lavaretus*	European whitefish	81	80
*Equus caballus*	Horse	254	245
*Gallus gallus*	Red Jungle Fowl	66	66
*Glyphis glyphis*	Speartooth Shark	94	94
*Hypophthalmichthys molitrix*	Silver carp	30	29
*Hypophthalmichthys nobilis*	Bighead carp	36	35
*Macaca fascicularis*	Crab-eating macaque	44	44
*Mus musculus*	mouse	53	50
*Mus musculus domesticus*	House mouse	59	59
*Myodes glareolus*	Bank vole	35	35
*Orcinus orca*	Killer Whale	87	87
*Ovis aries*	Sheep	94	94
*Pan paniscus*	Banobo	54	54
*Pan troglodytes schweinfurthii*	Eastern chimpanzee	33	33
*Pan troglodytes troglodytes*	Central chimpanzee	56	54
*Pan troglodytes verus*	Western chimpanzee	30	30
*Rattus norvegicus*	Brown Rat	66	66
*Sus scrofa*	Wild Boar	150	150
*Syncerus caffer*	African buffalo	45	45
*Tursiops truncatus*	Common bottlenose Dolphin	50	50
*Urocyon littoralis catalinae*	Island Fox	41	41
*Urocyon littoralis clementae*	Island Fox	33	33
*Urocyon littoralis santacruzae*	Island Fox	42	42
*Ursus arctos*	Brown Bear	74	74
*Ursus spelaeus*	Cave Bear (extinct)	34	20

### Sequence quality control

GenBank contains both curated and uncurated sequences from a large number of sources, which are of differing quality. A number of tests were applied to the sequences to assess the reliability of the data. (1) Sequence length of the mt-tRNA-Leu (UUR) genes extracted was compared to the length of the rCRS mt-tRNA-Leu (UUR) gene using the Biopython SeqIO module. Any sequences that were 5 nucleotides longer or shorter than the length of the rCRS mt-tRNA-Leu (UUR) gene were manually examined. (2) Unknown/unspecified bases (Ns), sequences with a high number of Ns are an indication of poor quality sequence data. The SeqIO module was used to determine the number of Ns with the mt-tRNA sequences. Any sequences which contained more than 5 Ns were removed from the study. (3) All of the mt-tRNA genes were aligned using ClustalW for each species and the statistics from these alignments was compiled. This file contained information about the number of sequences, the length of sequences within the file and a summary of the pairwise alignments scores. Pairwise scores are generated by ClustalW, before the full multiple sequence alignment, by comparing each pair of sequences to be aligned. They are a measure of the number of sequence identities divided by the sequence length. Mt-tRNA sequences from the same species should share a high degree of similarity and low similarity of sequences could be an indication of incorrect annotation, mislabelling of genes, or poor quality sequence data. The pairwise scores produced by ClustalW were used to highlight any sequences with a low degree of similarity to other sequences from the same species.

### tRNA secondary structure analysis

Two tools were used to study the secondary structures of tRNA genes. Alignments from this study were compared to the alignments in the tRNA-MAMIT [14] to determine the functional regions of the gene. “tRNAscan-SE Search Server” was used to predict the secondary structure of genes [35]. This software predicts the folding of tRNA molecules. The source was set to “Mito/ Chloroplast”.

### Phylogenetic analysis

Phylogenetic analysis, NETWORK version 4.6.1.3 was used to study the phylogenetic relationship of mt-tRNA-Leu (UUR) sequences [36].

## Results

### Disease associated mitochondrial m-tRNA-Leu variants found in vertebrates

**Fig 1:**
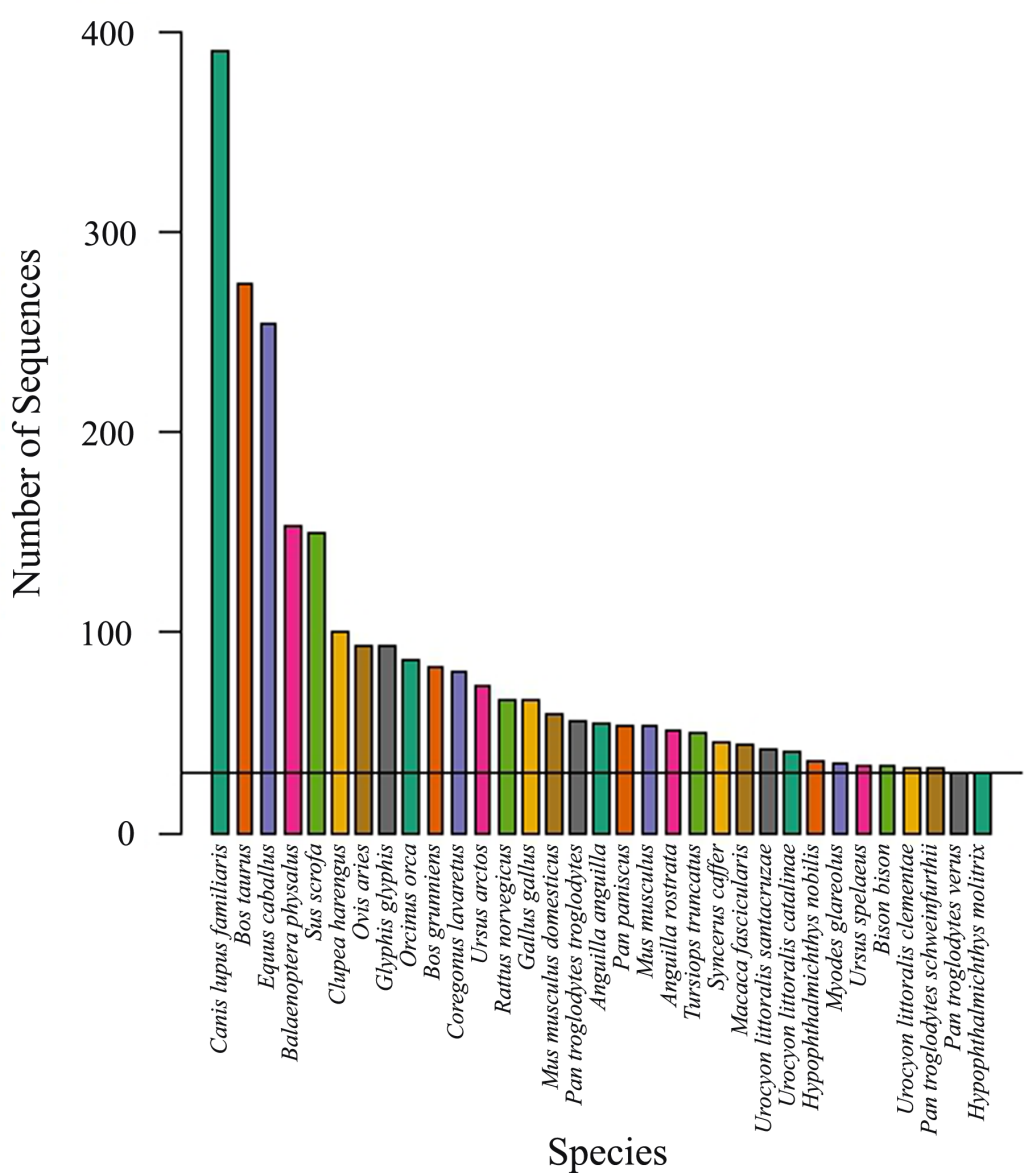
Species from the phylum Chordata with over 30 complete mitochondrial sequences.

The first part of this study considered all disease-associated mutations located on mt-tRNA-Leu (UUR) using a panel of species. mt-tRNA-Leu(UUR) was chosen, as it contains a number of clinically significant mutations including the most common mtDNA point mutation m.3243A>G. Initially 16,992 complete eukaryote (non-human) mitochondrial DNA genomes were downloaded from the GenBank database, and then all chordate sequences were extracted. A total of 33 species were selected for use in the study, with the majority being vertebrates, each with 30 or more complete sequences available (Fig 1). Details of species selection (Table 1), quality control (QC) applied to the sequences used and pipeline validation are described in the methods, with the detailed results of the QC being described in the supplemental data.

The restriction to species with 30 sequences allows us to assess the within-species variation critical to this study, as disease causing mutations will occur only at low frequencies. In total, 2784 sequences were available from the 33 species selected for use in the study. The sequences were derived from 406 independent studies. A total of 32 variants located on mt-tRNA-Leu (UUR) are reported to be associated with disease on either MITOMAP, tRNA-MAMIT or from a literature search. Therefore, we examined each of the 2784 sequences for any of these 32 variants.

**Fig 2:**
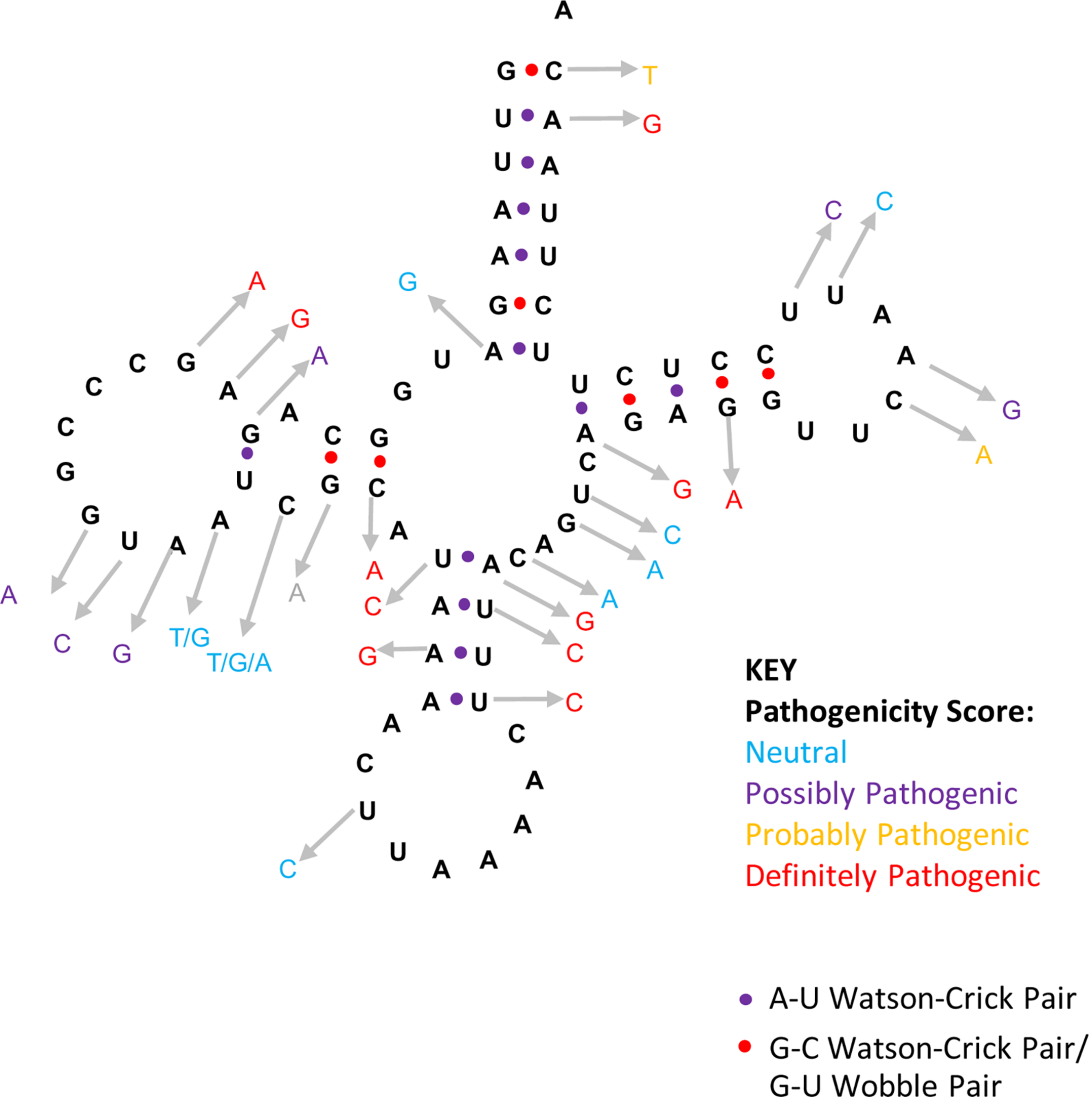
Classification of variants with reports of disease association within mt-tRNA-Leu (UUR) gene.

One potential problem is that the disease associations in the various online databases may not all be well supported by clinical and laboratory evidence [37]. So, we conducted an up-to-date literature search to assess the variants reported as pathogenic in humans using a scoring system applied widely in the mitochondrial field [32]. This system considers factors such as the number of times a mutation has been reported and evolutionary conservation. However, critical to its reliability is the emphasis on laboratory investigation of the proposed mutation. In particular, experiments that link genotype and phenotype, such as single muscle fibre analysis. After completing the re-evaluation of the mutations reported in the literature for mt-tRNA-Leu (UUR), 12 of the 32 reported mutations were designated as neutral polymorphisms, 8 were scored possibly pathogenic, 1 was scored probably pathogenic, while 11 scored as definitely pathogenic. The distribution of the human mutations on the mt-tRNA structure is shown Fig 2. These results support previous analysis of the data in these public databases [7, 32, 37]. This assessment was used to guide our interpretation of the data that we found in other species. In other words, to ensure that any disease casing variants seen in other species in the absence of disease are bona fide disease causing mutations.

Of the 32 known or suspected pathogenic variants located on the human mt-tRNA-Leu (UUR), 12 out of 32 were *monomorphic* in another species. That is present in all sequences from one (or more) non-human species. In Table 2, the variants considered to be definitely or probably pathogenic, after the application of the Yarham criteria described above, are highlighted. Six of these variants were located within stem regions of the mt-tRNA molecule and six within the loop or the variable regions. Of the six present in the stem regions, all showed a corresponding change in the other arm of the stem, so maintaining the Watson-Crick type pairing; and also the secondary structure. This is important because variants which disrupt Watson-Crick pairs in mt-tRNA molecules are frequently classed as pathogenic mutations [37]. Interestingly, the m.3251A>G mutation associated with mitochondrial myopathy seen in the mt-tRNA d-loop [38, 39] was present in all 74 sequences from *Ursus arctos* (brown bear) and all 20 sequences from *Ursus spelaeus* (cave bear). This mutation corresponds to base 22 in mt-tRNA-Leu(UUR); it is involved in a long range tertiary interaction affecting the following triplet (13–22)-46 [21]. The *Ursus arctos* and *Ursus spelaeus* sequences also differ at base 46, with a change of a C to T changing the nature of the tertiary structure [21] and potentially acting as a compensatory change.

**Table 2:** SNPs which are known or suspected to be pathogenic in humans which are seen in 100% of sequences from other species, positions shown equates to location within rCRS sequence.

Position	Region	Variant	Status	Secondary Structure	Tertiary Structure	Species
3236 (Bosley *et al*., 2008)	acc stem	A-G	Neutral			All non primate species
3250 (Goto *et al*., 1992)	d-loop	T-C	Possibly Pathogenic	N/A	(8–14)-21	*Glyphis glyphis*, *Orcinus orca*, *Gallus gallus*, *Tursiops truncatus*
3251 (Sweeney *et al*., 1993)	d-loop	A-G	Possibly Pathogenic	N/A	(13–22)-46G–A>G–C>T	*Ursus arctos*, *Ursus spelaeus*
3254 (Chen *et al*., 2000)	d-stem	C-T	Neutral	A-C mismatchto A-T pair	(25–10)-45	All species except *Pan paniscus*
3264 (Suzuki *et al*., 1997)	ac-loop	T-C	Neutral	N/A		*Glyphis glyphis*, *Coregonus lavaretus*, *Anguilla anguilla*, *Anguilla rostrata*, *Hypophthalmichthys nobilis*, *Hypophthalmichthys molitrix*
3271 (Goto *et al*., 1991)	ac-stem	T-C	Definitely Pathogenic	A-T pairto C-G pair		*Glyphis glyphis*, *Coregonus lavaretus*, *Rattus norvegicus*, *Gallus gallus*, *Mus musculus domesticus*, *Anguilla anguilla*, *Anguilla rostrata*,*Mus musculus*, *Hypophthalmichthys nobilis*, *Hypophthalmichthys molitrix*,*Myodes glareolus*
3273 (Campos *et al*., 2001)	ac-stem	T-C	Definitely Pathogenic	A-T pairto C-G pair	26-44No Change	*Clupea harengus*
3275(Garcia-Lozano *et al*., 2000)	Variable Region	C-A	Neutral	N/A	(13–22)-46	*Hypophthalmichthys nobilis*, *Hypophthalmichthys molitrix*
3290(Zhu *et al*., 2009)	t-loop	T-C	Neutral	N/A		*Equus caballus*, *Sus scrofa*, *Anguilla anguilla*, *Anguilla anguilla*, *Anguilla rostrata*, *Macaca fascicularis*, *Urocyon littoralis santacruzae*, *Urocyon littoralis catalinae*, *Urocyon littoralis clementae*
3291(Goto *et al*., 202AD)	t-loop	T-C	Possibly Pathogenic	N/A		*Coregonus lavaretus*
3302 (Bindoff *et al*., 1993)	acc stem	A-G	Definitely Pathogenic	A-T pairto C-G		*Clupea harengus*, *Coregonus lavaretus*, *Gallus gallus*, *Hypophthalmichthys nobilis*, *Hypophthalmichthys molitrix*
3303(Silvestri *et al*.)	acc stem	C-T	Probably Pathogenic	A-T pairto C-G		*Ursus spelaeus*, *Sus scrofa*, *Rattus norvegicus*, *Myodes glareolus*, *Mus musculus domesticus*, *Mus musculus*, *Hypophthalmichthys nobilis*, *Hypophthalmichthys molitrix*, *Clupea harengus*

We observed a number of the variants associated with specific species or taxonomic groups such as the m.3264T>C, m.3271T>C, m.3273T>C, m.3275 C>A, m.3302A>G, which were all confined to species of fish. Other such observations include the occurrence of the m.3271T>C variant in fish and rodents; similarly, mutations are often seen in parallel branches of the human phylogeny, probably due to mtDNA’s high rate of mutation [40]. The m.3254C>G SNP was monomorphic in all species except *Pan paniscus* (bonobo), which had a C at this position, and the m.3226A>G SNP was monomorphic in all non-primate species.

We found an additional four disease associated variants in other species, but here there were two polymorphisms at the sites in sequences of the species in question (Table 3).

**Table 3:** The frequency of known/ suspected pathogenic SNPs which are polymorphic in other species (Position shown equates to location within rCRS genome sequence).

Base	Region	Variant	Status	Species	No. Sequences
3243 (Goto *et al*., 1990)	D-Loop	A>G	Definitely pathogenic	*Canis lupus familiaris*	57/391
3244 (Mimaki *et al*., n 2009)	D-Loop	G>A	Definitely pathogenic	*Ursus arcto*	2/72
3249 (Seneca *et al*., 2001)	D-Loop	A>G	Possibly Pathogenic	*Sus scrofa*	1/150
3290 (Zhu *et al*., 2009)	T-Loop	T>C	Neutral	*Canis lupus familiaris*	2/391
3290 (Zhu *et al*., 2009)	T-Loop	T>C	Neutral	*Pan paniscus*	1/54

- The m.3243A>G located in the d-loop of the mt-tRNA molecule, is the most common point mutation associated with mitochondrial disease in humans. The m.3243A>G mutation was seen in 57 out of 391 sequences from *Canis lupus familiaris* (dog) [29].
- The m.3244G>A mutation also located in the mt-tRNA’s d-loop, was seen in 2 out 72 sequences from *Ursus arctos* (HQ685945, HQ685940). Both sequences were obtained from a study of migration patterns among brown bears. The m.3244G>A mutation has been observed in a patient with MELAS [41] and using the pathogenicity scoring criteria [7] it is classed as definitely pathogenic in humans.
- The m.3249G>A mutation, which is located in the mt-tRNA’s d-loop, was seen in 1 in 150 *Sus scrofa* (Wild boar) sequences (DQ268530). This mutation was first detected in 2001 in one patient with a clinical phenotype resembling Kearns-Sayre syndrome [42, 43]. However, it was only designated as possibly pathogenic using the scoring system of Yarham et al 2011 [32].
- The m.3290T>C mutation was seen in 2/391 sequences from *Canis lupus familiaris* and 1/54 *Pan paniscus* sequences. When this mutation was investigated, it was also found to be polymorphic in humans, being seen in multiple populations, and with limited evidence existed linking it with disease. It has, therefore, been classified as neutral [32].

### Differences in secondary structure of Mt-tRNA-Leu (UUR) with the m.3243A>G in dog (*Canis lupus familiaris*)

**Fig 3:**
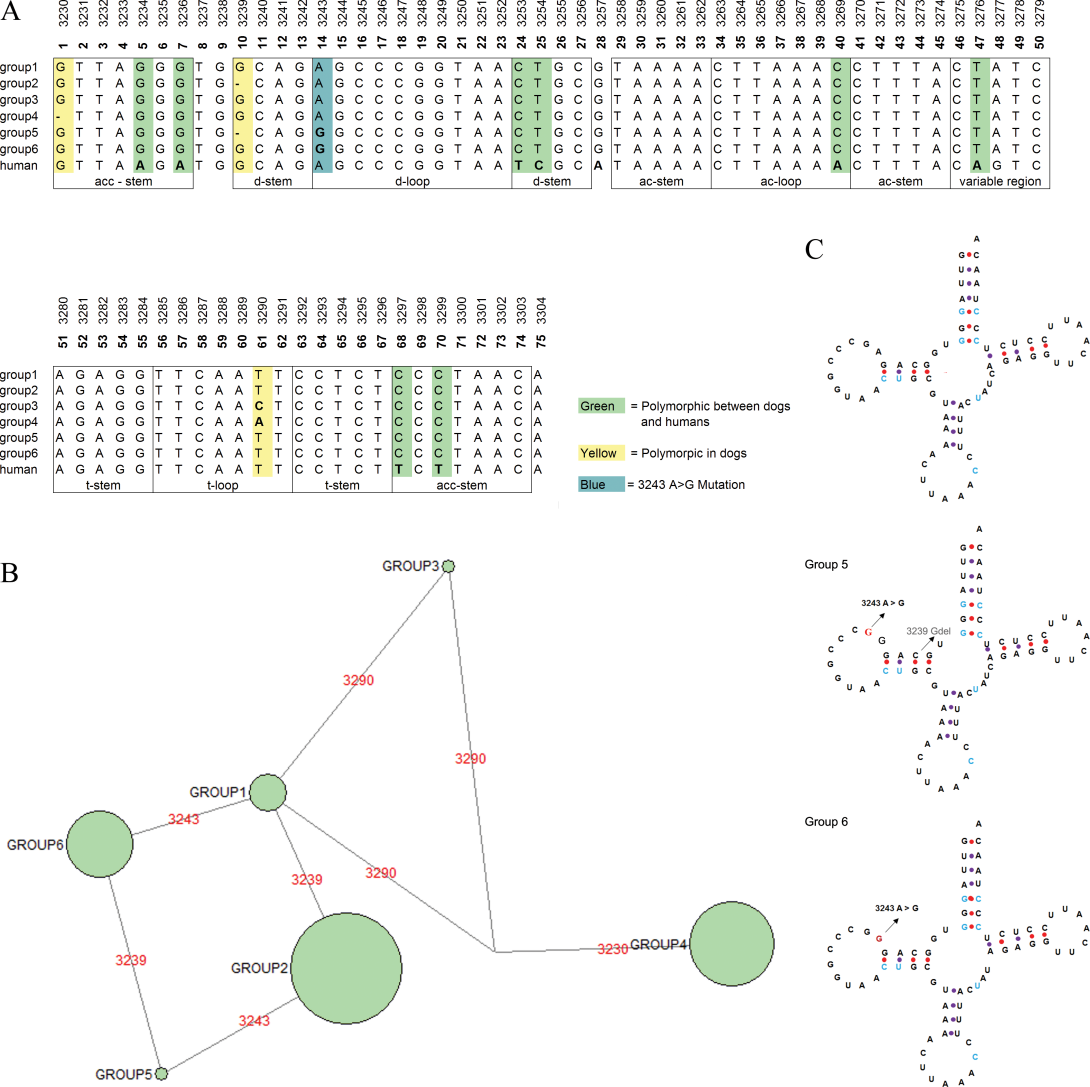
(A) Alignment of 6 unique mt-tRNA-Leu (UUR) sequences from *Canis lupus familiaris* with the rCRS. Reticulation indicates one of the variants observed at positions 3239, and 3243 has occurred more than once in *Canis lupus familiaris*. (B) A phylogenetic network, created using *Canis lupus familiaris* mt-tRNA-Leu (UUR) sequences. The first structure shows the positions of polymorphisms between all of the sequences studied from *Canis lupus familiaris* and humans. The second two structures show the tRNA molecule where the 3243 A>G polymorphism was observed in *Canis lupus familiaris*. (C) The secondary structure of the mt-tRNA-Leu (UUR) from *Canis lupus familiaris*.

The largest number of sequences available for a single group was from the dog (*Canis lupus familiari*) with a total of 391 sequences. All available sequences from the dog were aligned with the human mt-tRNA-Leu gene. The resulting alignment was 75bp in length, which corresponds to the entire length of the mt-tRNA-Leu(UUR) gene. The dog mt-tRNA-Leu(UUR) sequence is divergent from the human reference sequence at eight locations, shown in green on the alignment (Fig 3A). The m.3243A>G mutation was found in 57 out of 391 alignments of dog mt-tRNA-Leu(UUR). Prior work has suggested that this variant was fixed in dogs, this work was conducted when the available sequence data was more limited than today [30] the availability of clades with and without the m.3243A>G mutation makes for a more powerful model to investigate possible compensatory changes. In addition to the m.3243A>G mutation, there are three other positions within the mt-tRNA-Leu(UUR) which where the sequences were divergent from human mt-tRNA-Leu gene.

As mentioned, the presence of dog sequence with and without the well-studied m.3243A>G mutation has allowed a more detailed investigation into possible compensatory effects than was possible previously [30]. The polymorphisms were used to create a phylogenetic network that contained six haplogroups (Fig 3B), using NETWORK [36]. The haplotypes used are shown in (Table 4). The largest group was haplogroup 1 containing 328 sequences, which was 83% of the total. The other groups most likely evolved from haplogroup 1, as larger groups are believed to have a higher likelihood of being ancestral [36]. Two deletions were observed in position 3230 and 3239, seen in groups 4 and groups 2 and 5 respectively. The m.3290T>A variant was observed in group 2 and m.3290T T>C was observed in group 4. The m.3243A>G mutation was confined to two haplogroups (haplogroup 6 and haplogroup 5). We postulated alterations to the secondary structure of the mt-tRNA-Leu(UUR) supress the pathogenicity of the m.3243A>G mutation. To address this, we analysed the secondary structure of sequences containing the m.3243A>G polymorphism. Two tools were used to study the secondary structures of the gene: we compared alignments to those from tRNA-MAMIT [14] to determine the functional regions of the gene; and “tRNAscan-SE Search Server, was used to predict the secondary structure of genes [35]. The predicted secondary structures of the three mt-tRNA-Leu (UUR) dog haplotypes are shown in (Fig 3C).

**Table 4:** The frequency of key SNPs on the *Canis lupus familiaris* phylogeny (Position shown equates to location within rCRS, human mtDNA reference sequence).

	Character				
Sequence	3230	3239	3243	3290	Frequency
Group 1	G	G	A	T	328
Group 2	G	-	A	T	3
Group 3	G	G	A	C	2
Group 4	-	G	A	A	1
Group 5	G	-	G	T	2
Group 6	G	G	G	T	55

Two positions, divergent from the human sequence, and within the D-Stem of the dog sequences are particularly interesting. First, m.3253T>C, as all of the sequences have a C at position 24 of the mt-tRNA-Leu molecule, whereas the human sequence has T at the same position. This corresponds to the final base in the D- Stem and changes a wobble pair into a Watson-Crick pair. This polymorphism was only seen in 8 other sequences included in this study, all from *Macaca fascicularis* (crab-eating macaque). Second m.3254 C>T, as there is T at position 25 within the molecule instead of a C creating an extra Watson-Crick pair in the d-stem of the dog mt-tRNA-Leu molecule. Both of these variants change the secondary structure of the molecule and we hypothesise that they could suppress the pathogenicity of the m.3243A>G mutation.

The sequences from dog are divergent from the human sequence in six other locations. Four of these changes are in the acc-stem maintaining Watson-Crick base pairings but changing the pairs from A-T in humans to G-C in *Canis lupus familiaris*. A further difference is located in the variable region, as well as the T- loop. These six variants maintain and do not alter the secondary structure of the molecule and so were not studied further.

### The distribution of predicted compensatory variants, identified in *Canis lupus familiaris*, in other vertebrates

One possible reason for the presence of mutations that are human disease-associated, in species other than humans is that these mutations are compensated for; therefore, we conducted an extended study of the variants m.3254C>T and m.3253T>C, which may compensate for the m.3243A>G mutation. The investigation was extended by studying m.3254C>T, m.3253T>C where it co-occurred with the m.3243A>G in 10,426 GenBank records from vertebrates. The m.3254C>T and m.3253T>C variants are interesting as they change the secondary structure of the mt-tRNA molecule and are present in all sequences containing the m.3243A>G in *dog*. The deletion m.3239G>- was not studied further because it was only observed in 3.5% of the sequences, belonging to phylogenetic group 5 (see Fig 3C), which contain the m.3243A>G mutation and the network analysis suggested that it was acquired after the m.3243A>G. The m.3254C>T polymorphism was within 97% of the vertebrate species sequences analysed with an even distribution amongst taxonomic groups. The m.3254C appears to be the most common genotype in the majority of vertebrate species studied.

Considering next the m.3253T>C polymorphism within other vertebrate, it was observed within 15 out of 139 orders/suborders/superorders studied (Table 5). It was present with the highest frequency within the taxonomic order *Proboscidea*, where it was found in all 37 sequences analysed. Additionally, it was observed with a frequency of 0.436 within the order *Carnivora*, which contains canid species. Within the family *Canidae*, m.3253T>C is present in 421 out of 631 sequences. It is present in 100% of sequences from sub species of *Canis lupus* and in 1/5 *Canis latrans* (coyote) sequences (Table 6).

**Table 5:** The number of sequences containing the m.3253T>C polymorphism and the frequency of the occurrence within vertebrata groupings.

Order	Present	Total Sequences	Frequency
Proboscidea	37	37	1.000
Carnivora	438	1005	0.436
Pholidota	4	11	0.364
Primates	50	604	0.237
Neoteleostei	320	1886	0.170
Acipenseriformes	2	33	0.061
Perissodactyla	9	299	0.030
Chiroptera	2	87	0.023
Cryptodira	3	150	0.020
Squamata	7	379	0.018
Caudata	2	186	0.011
Anguilliformes	2	217	0.009
Ostariophysi	9	1261	0.007
Rodentia	2	454	0.004
Cetartiodactyla	2	1599	0.001

**Table 6:** Percentage of sequences carrying the 3243A>G and 3253T>C SNP within the order carnivora.

Family	Species	3243A>G	3253A>C
Canidae	*Canis lupus familiaris*	15	100
Canidae	*Canis lupus*	0	100
Canidae	*Canis lupus campestris*	0	100
Canidae	*Canis lupus desertorum*	0	100
Canidae	*Canis lupus lupus*	0	100
Canidae	*Canis lupus chanco*	0	100
Canidae	*Canis lupus laniger*	0	100
Canidae	*Canis latrans*	0	20
Phocidae	*Lobodon carcinophaga*	100	100
Phocidae	*Hydrurga leptonyx*	0	100
Phocidae	*Leptonychotes weddellii*	100	100
Phocidae	*Mirounga leonina*	0	100
Ursidae	*Ursus thibetanus*	0	8
Felidae	*Puma concolor*	0	100
Felidae	*Felis catus*	0	100
Felidae	*Acinonyx jubatus*	0	100

**Fig 4:**
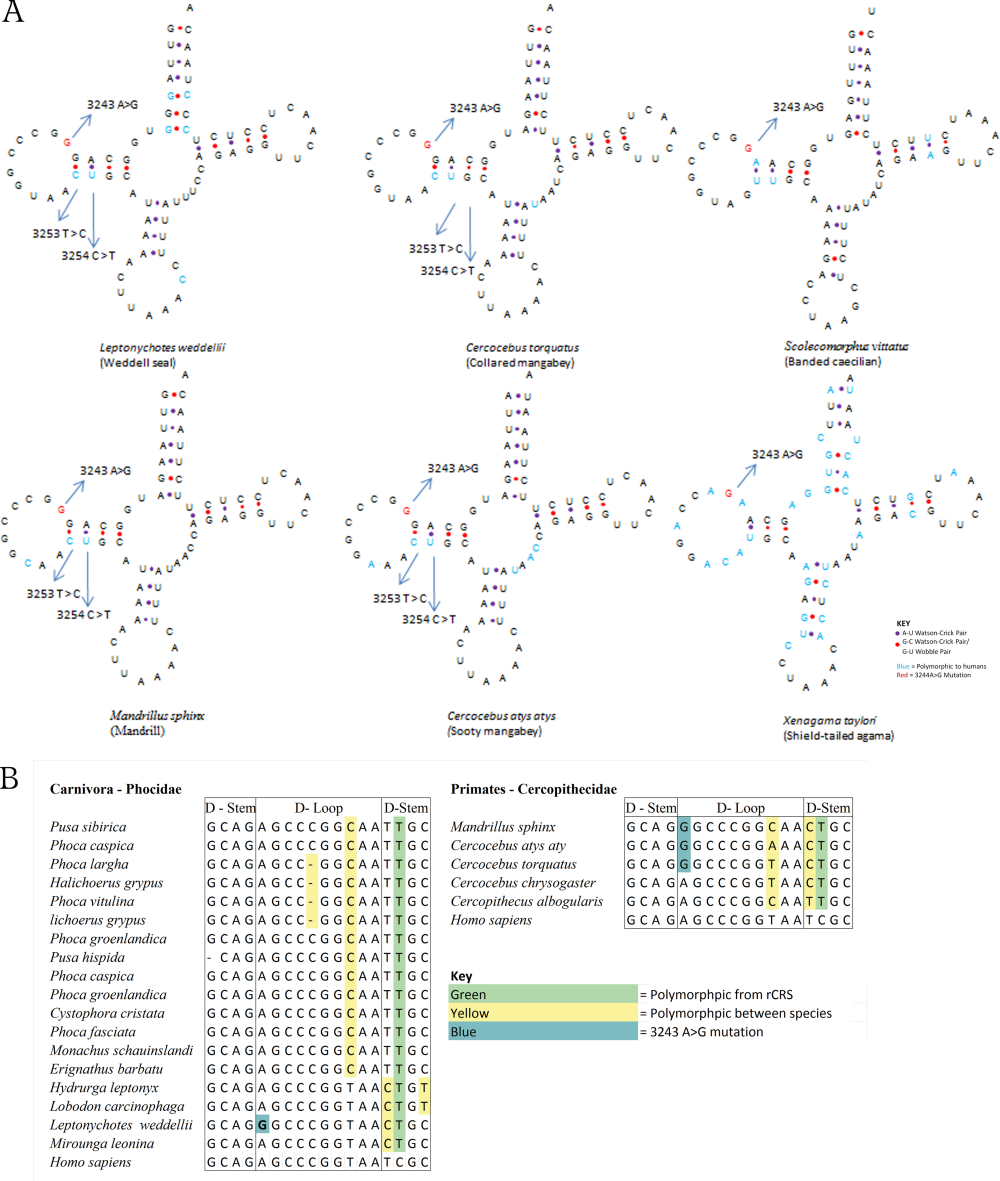
(A) The secondary structure of mt-tRNA-Leu molecules carrying the 3243 A>G mutation. (B) Alignments of tRNA’s D-Loop of from carnivore and primates carrying the 3243 A>G mutation.

The m.3253T>C variant is also present in the families *Phocidae*, *Felidae* and *Urisdae* with frequencies of 25%, 21% and 0.6%. The m.3243A>G mutation was found along with m.3253T>C mutation in 2 sequences from the species *Leptonychotes weddellii* (weddell seal), which belongs to a taxonomic family of earless seals and *Phocidae* within the order *Carnivora* (Fig 4). Only two sequences were available for the species *Leptonychotes weddellii*. A total of 32 sequences were analysed from the family *Phocidae*, with the m.3253T>C mutation present in 8 of these sequences. All of species within *Phocidae* contained the m.3254C>T, which provides an additional Watson-Crick pairing in the d-stem of the mt-tRNA-Leu(UUR) molecule that is not seen in humans. An additional polymorphism was present at position m.3256C>T in 2 sequences from *Hydrurga leptonyx* (leopard seal) and 2 sequences from *Lobodon carcinophaga* (crabeater seal); this decreases the number of Watson-Crick pairs in the mt-tRNA-Leu(UUR) d-stem in these species. Additionally, a total of 604 mt-tRNA-Leu(UUR) sequences from primate species were analysed. The m.3243A>G mutation along with m.3253T>C variant were found in three species of old world monkeys within the family *Cercopithecidae*. In the species *Mandrillus sphinx*, these SNPs were present in all 3 of the sequences available for the species (KJ434963.1, NC_021956.1, KC757403.1). Both SNPs were also observed in the only sequence from (KP090062.1) and in the only 2 sequences available for *Cercocebus torquatus* (white collared mangabey) (NC_023964.1, KJ434959.1).

The m.3243A>G mutation was found in 2 sequences from *Scolecomorphus vittatus* (AY456253.1 and NC_006304.1), a legless amphibian. The sequences came from different sources but are identical. The ClustalW pairwise score between the human and *Scolecomorphus vittatus* sequences within the alignment is 73.33 indicating a low degree of similarity between the gene sequences. The pairwise score is a measurement of the number of identities in a pair of sequences divided by the length of the sequence, and is expressed as a percentage. Within the alignment the *Scolecomorphus vittatus* sequence differs from the human reference genome in 20 out of 75 nucleotides, with 13 of these polymorphisms occurring in the stem regions of the mt-tRNA molecule. The m.3253A>C variant is not present in this sequence. However, when the secondary structure was examined, the tRNA d-Stem contained four Watson-Crick pairs as opposed to the two that are found in human mt-tRNA-Leu(UUR). Two sequences from *Xenagama taylori* (NC_008065.1 and DQ008215.1) the shield-tailed agama contained the 3243A>G. The two sequences were 73 bp in length and were identical. They were the only available sequences for this species. The sequences were highly divergent from the rCRS with a pairwise score of 56.16%. There were a number of changes in the secondary structure: the d-stem was only 3bp and the ac-stem contained an additional nucleotide and was 5bp in length.

The evidence gathered from current databases shows there are variants that have the potential to compensate for mutations known to cause disease in humans, and that the spread of these variants is such that there is unlikely to be a universal list of disease causing variants across different species, and even within a species.

### The distribution of predicted compensatory variants in other humans

Finally, we have investigated directly whether compensatory mutations are present in human mtDNA present in the public databases. The m3243A>G, m.3253T>C and m.3254C>T variants were also studied within human mitochondrial sequences present on the public databases. Out of a total of 30,524 complete human mitochondrial sequences downloaded from GenBank, the m.3243A>G mutation was observed in 8 sequences. Unsurprisingly, 7 of these sequences were derived from sequences described as patient data. The 3243A>G mutation was also present in another human sequence KJ185483.1, which was derived from a population study, and there is no evidence to confirm whether or not this individual showed any disease symptoms. No potential compensatory mutations were observed in this sequence.

Distribution of the putative m.3253 T>C and m.3254C>T putative compensatory variants in humans was investigated, using the MITOMAP database of mitochondrial sequence variants derived from 29,867 complete human mitochondrial sequences present in GenBank in December 2014. Within these sequences, the m.3253T>C occurs in 7 sequences with a frequency of 0.02% and the m.3254C>T variant is present in 9 sequences with a frequency of 0.03%. The sequences that contained the m.3253T>C mutations were derived from 7 independent studies. Of these sequences, 3 belonged to the haplogroup M10a1, 3 belonged to haplogroup L2 and one belonged to haplogroup U6a3 (Table 7). Using information about haplogroup frequencies contained within MITOMAP the m.3253T>C variant was found to occur at the highest frequency within the L2 haplogroup. Although the numbers of human sequences bearing the m.3253 T>C and m.3254C>T are relatively low, their presence supports the need to consider sequence context when making decisions about the pathogenicity of variants in the human mt-tRNA’s. It also suggests that, where known disease causing variant are seen in novel phylogenetic contexts with unexpected presentations, it would be of interest to conduct a number of the gold standard laboratory analysis [32] used to link genotype-phenotype to ensure the known disease casing mutation is causing disease in this context.

**Table 7:** Sequences that carry the 3253 point mutation.

Sequence ID	Haplogroup	Percentage within haplogroup
KP702293.1	U6a3	0.04
KF451676.1	M10a	0.11
KJ446421.1	M10a	0.11
JQ045037.1	L2b	0.35
JQ044890.1	L2b	0.35
JN857060.1	M10a	0.11
DQ112702.2	L2b	0.35

### Disease associated variants from all mitochondrial m-tRNA studied in vertebrates

**Fig 5:**
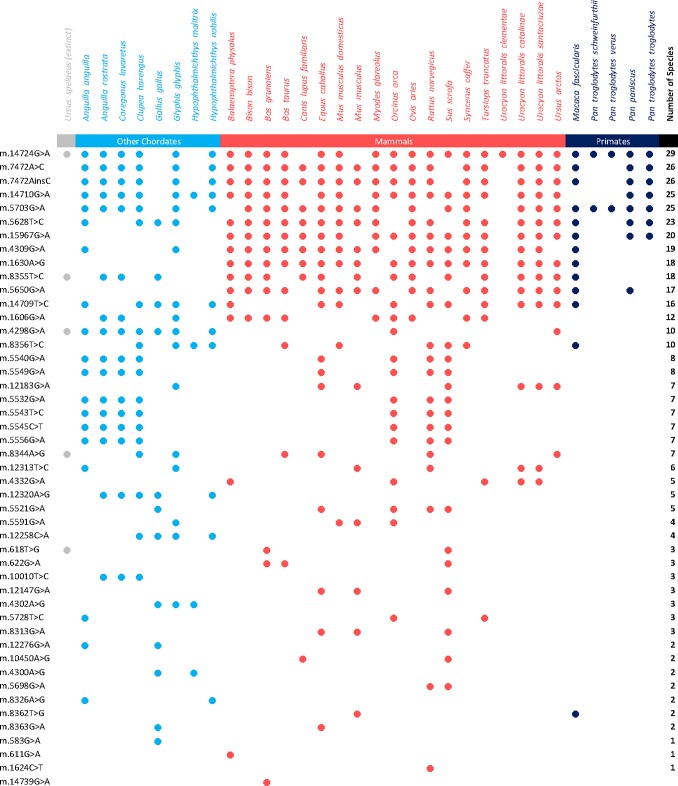
Mutations which are classified as definitely pathogenic in humans that are found to be fixed in other species.

We concluded the study by determining whether the results we had seen in mt-tRNA-Leu(UUR) were specific to this tRNA molecule or whether the trend would be replicated in other mt-RNAs. We selected a total of 246 disease asssociated variants in the remaining 21 m-tRNA molecules and looked for their presence or absence in the original panel of 33 species. Only 4% of these variants were not observed in any species and the remaining 235 mutations were observed in at least one species. This panel of mutations was compared to data published by Yarham et al., 62 of these mutations had been previously classified as definitely pathogenic in humans were studied further [32]. Of the definitely pathogenic mutations 47 were seen to be monomorphic for at least one species (Fig 5). These numbers differ to those in a prior report [30] as we applied a clinically validated scoring system [3, 7, 32] to ensure that we look at variants defiantly associated with clinically manifesting disease this algorithm was not available prior to the first report being published.

Most of these definitely pathogenic mutations were only monomorphic in less than 10 of the species studied. However, but there were a number of mutations which were found in the majority of species studied. Notably, G5703A, G14710A, A7472C, and G14724A, were observed in more than 75% of the species studied.

## Discussion

There have been many discussions about the causes of the variable presentation of mitochondrial disease. One possible much discussed explanation for this is the wide sequence context. The unique inheritance pattern of mtDNA results in the emergence of distinct maternal lineages or haplogroups. This has the effect that once a *compensatory* mutation, has occurred a subsequent normally pathogenic mutation can occur on the lineage and spread. This leads to the possibility that some lineages might be more robust than others to the consequences of mutation. Public databases now contain a wealth of sequence information from individuals, both human and other species. Prior studies that have considered this question have been limited by availability of sequence data, with some studies only using a single sequence form each of the species considered [31]. We have used a greatly expanded sequence database to investigate the prevalence and penetrance of variants located in the gene for mt-tRNA-Leu (UUR). This is a known location of disease-causing mutations in humans. Supporting the idea outlined above, we have found a number of widely recognized human mutations in other species at high frequencies, and have been able to suggest compensatory mutations.

### Observed secondary structure variation in tRNA-Leu(UUR) molecule in species with 3243A>G mutation

In this study, we show that m.3243A>G mutation occurs at high frequency within sequences from 6 species *Canis lupus familiaris*, *Mandrillus sphinx*, *Cercocebus atysatys*, *Cercocebus torquatus*, *Leptonychotes weddellii*, *Scolecomorphus vittatus* and *Xenagama taylori*. This suggests that the mutation is not pathogenic within these species. This is especially likely with samples taken from wild animals, which are under intense selection. In *Canis lupus familiaris*, the m.3243A>G mutation is present in approximately 15% of sequences. While only limited data is available for *Mandrillus sphinx*, *Cercocebus atysatys*, *Cercocebus torquatus*, *Leptonychotes weddellii*, *Scolecomorphus vittatus* and *Xenagama taylori*, the variant is present in 100% of the available sequences. Although the m.3243A>G nucleotide is not present within a stem region of the mt-tRNA-Leu(UUR) molecule, it is involved in a tertiary interaction between bases 8, 14 and 21 of the mt-tRNA-Leu [21]. Disruption to the 3D structure of the molecule is believed to contribute to the pathogenic effect of the mutation [25].

In the current study, we found possible compensatory mutations, which alter the secondary structure of the mt-tRNA-Leu(UUR) molecule. These mutations may affect the way the mt-tRNA molecule folds and negate the negative effects of m.3243A>G. The compensatory mutations, observed in the d-stem of the mt-tRNA-Leu(UUR) molecule, were the m.3253A>C and m.3254C>T SNPs. They were present along with the m.3243A>G mutation in *Canis lupus familiaris*, *Mandrillus sphinx*, *Cercocebus atysatys*, *Cercocebus torquatus* and *Leptonychotes weddellii*. The d-stem of the human mitochondrial mt-tRNA-Leu(UUR) gene is 4 nucleotides long. It contains 2 Watson-Crick pairs, two nucleotides which are unpaired and a G-U wobble base pair in the position adjacent to the d-loop. The two compensatory mutations create a Watson-Crick base pair in the d-Stem of the mt-tRNA molecule in the position of the Wobble pair, and an extra Watson-Crick pair in the place of the two unpaired nucleotides. Changes to the secondary structure may suppress the pathogenic effect of the m.3243A>G mutation by altering the 3D shape of the mt-tRNA molecule in two ways: (1) Wobble pairs have been shown to change the 3D structure of mt-tRNA molecules due to the fact that G-U pairs form different glycosidic pairings to Watson-crick pairs. This alters the angle of the bond with respect to the backbone of the molecule resulting in changes to the 3D shape of the molecule; (2) G-U pairs display conformational flexibility. These pairings react more sharply to sequence context than Watson-Crick pairs. The twisting in the molecule is influenced by the identity of the base pairs immediately adjacent to the wobble pair. As the m.3243A>G is directly adjacent to the wobble base pair, it possibly causes the molecule to twist in a way that prevents the correct 3D structure forming [44]. The tRNA-Leu(UUR) molecule of *Xenagama taylori* is significantly different from the rCRS and other mt-tRNA-Leu(UUR) molecules studied here. The reduced length position in d-stem means that the base identified as m.3243A>G is actually the second nucleotide in the d-loop rather than the first. Therefore, it is difficult to make direct comparisons about the structural importance of this base change.

The m.3254T>C mutation appears to be the most common allele and is prolific throughout the species studied, whereas the m.3253T>C mutation is confined to specific taxonomic groups. Within carnivores the m.3253T>C is seen in related species of wolf, dog and dog-like species, but no other closely related species. This indicates that the mutation was acquired after divergence of the canids. Variants which occur in regions that are highly conserved across species are most likely to be pathogenic and disease is most likely to be associated with rare variants. The wide distribution of these variants indicates that they are neutral mutations.

### Evidence that haplogroup background may suppress expression of disease

It has previously been reported that mitochondrial diseases from patients belonging to the African haplogroup L do not show the same phenotypic expression of disease as patients with European haplogroups [8, 9, 16]. One instance of the m.3243A>G mutation was detected in human from a population study where no link to disease was reported [45]. The m.3253T>C variant, which appears to be a compensatory mutation, is seen with the highest frequency in sequences from the African haplogroup L, and the Asian haplogroup M, was seen on 3 sequences from each haplogroup. Whereas the m.3254T>C is seen with highest frequency in sequences from the European haplogroup J, and was seen in five sequences from the sub-haplogroup J1b. Secondary mutations which are specific to certain haplogroups have been shown to play a role in the phenotypic expression of mitochondrial disorders [17, 46]. It may be that some population variants are neutral on their own, but, when combined with a second mutation they can increase the severity of a disease [47].

The evidence presented here strongly indicates that changes to secondary structure of the mt-tRNA-Leu(UUR) molecule prevent pathogenic effects of 3243A>G. This supports the idea that sequence context (haplogroup background) is one of the important factors in the expression on mtDNA diseases. However, it must be remembered the phenotypes resulting from the m.3243A>G mutation are varied, and debate still exists as to its mechanism of action. The 3243A>G may impair methylation of mt-tRNA in position 10 of the molecule [21]. The presence of the mutation decreases the methylation of a uracil molecule in the first wobble position of the anticodon. This leads to a deficiency in the molecule in decoding UUG codons. Studying the methylation patterns of mt-tRNA molecules with additional mutations could provide information about how the mutation affects functionality. Neutral mutations elsewhere in other mitochondrial genes may supress the 3243 A>G mutation [20]. In humans, the tRNA-Leu(UUR) gene is adjacent to the 16S ribosomal RNA gene. The m.3243A>G mutation within the tRNA-Leu(UUR) gene may interfere with a transcription and termination site for the 16S RNA molecule [20], leading to an accumulation of unprocessed RNA. In the *Canis lupus familiaris* and *Leptonychotes weddellii* sequences, including those possessing the m.3243A>G mutation, the locations of mt-tRNA-Leu(UUR) gene and the16S ribosomal RNA gene are the same as the human mitochondrial genome and the transcription termination site is unchanged.

Previous research suggests Watson-Crick pairings are important in correct functioning of tRNA molecules and SNPs which break these bonds are more likely to be pathogenic [37, 48]. Evidence obtained in this study supports this hypothesis. There were three disease causing variants, with good evidence of pathogenicity within the stem regions of the tRNA molecule that were observed in 100% of sequences from non-humans, which strongly indicated that these mutations are not pathogenic within the species in question. In all instances, these mutations were accompanied by a compensatory change on the other arm of the stem of the tRNA molecule. This observation supports the previous hypothesis that it is not the SNP itself that is linked to disease but, rather, the disruption to the Watson-Crick pairs in these regions.

We identified a further 47 variants from other tRNA molecules which are classified as “defintitely pathogenic” in 100% of sequences from other species. Again this provides strong evidence that variations within the molecules of these species prevent the pathogenic effects that have been seen in humans.

To conclude, evidence does exist to support the hypothesis that animals can be affected by mitochondrial disease, and as such they represent a valid system for considering the penetrance of mtDNA mutations and the importance of lineage context [29]. Notably, the results here strongly suggest that the m.3243A>G not seen to be present at high levels in humans in the absence of disease, is present as a non-pathogenic variant in other species, and that mtDNA sequence context is key to the modulation of the impact of this mutation. The importance of sequence context has previously been considered in the context of mutations causing Leber's hereditary optic neuropathy (LHON) [17, 18]. As we sequence more, and investigate disease in more lineages [9], we are likely to find out more about the importance of sequence context in the expression of mtDNA variants. This knowledge will impact on the study of the role of mtDNA variants in clinical disease, and how we investigate any role of mtDNA variation in common complex diseases [49].
